# Patterns and determinants of health care utilization among people with Parkinson’s disease: A population-based analysis in Ontario, Canada

**DOI:** 10.1371/journal.pone.0305062

**Published:** 2024-06-21

**Authors:** Eric J. Crighton, Alexandra M. Ouédraogo, M. Sawada, Tiago A. Mestre

**Affiliations:** 1 Department of Geography, Environment and Geomatics, University of Ottawa, Ottawa, Ontario, Canada; 2 ICES, Ottawa, Ontario, Canada; 3 Laboratory for Applied Geomatics and GIS Science (LAGGISS), Department of Geography, Environment and Geomatics, University of Ottawa, Ottawa, Ontario, Canada; 4 Parkinson Disease and Movement Disorder Clinic, Department of Medicine, Division of Neurology, The Ottawa Hospital Research Institute, University of Ottawa, Ottawa, Ontario, Canada; University of Auckland, NEW ZEALAND

## Abstract

In Ontario, despite the increasing prevalence of Parkinson’s disease (PD), barriers to access-to-care for people with Parkinson’s disease (PwP) and their caregivers are not well understood. The objective of this study is to examine spatial patterns of health care utilization among PwP and identify factors associated with PD-related health care utilization of individuals in Ontario. We employed a retrospective, population-based study design involving administrative health data to identify PwP as of March 31, 2018 (N = 35,482) using a previously validated case definition. An enhanced 2-step floating catchment area method was used to measure spatial accessibility to PD care and a descriptive spatial analysis was conducted to describe health service utilization by geographic area and specialty type. Negative binomial regression models were then conducted to identify associated geographic, socioeconomic, comorbidity and demographic factors. There was marked spatial variability in PD-related service utilization, with neurology and all provider visits being significantly higher in urban areas (CMF>1.20; p<0.05) and family physician visits being significantly higher (CMF >1.20; p<0.05) in more rural areas and remote areas. More frequent visits to family physicians were associated with living in rural areas, while less frequent visitation was associated with living in areas of low spatial accessibility with high ethnic concentration. Visits to neurologists were positively associated with living in areas of high spatial accessibility and with high ethnic concentration. Visits to all providers were also positively associated with areas of high spatial accessibility. For all outcomes, less frequent visits were found in women, older people, and those living in more deprived areas as years living with PD increased. This study demonstrates the importance of geographic, socioeconomic and individual factors in determining PwP’s likelihood of accessing care and type of care provided. Our results can be expected to inform the development of policies and patient care models aimed at improving accessibility among diverse populations of PwP.

## Introduction

Parkinson Disease (PD) is a non-curable, neurodegenerative disorder affecting over 100,000 Canadians and 46,000 Ontarians, primarily over the age of 40 years, in 2021 [1]. Globally, the prevalence of PD is increasing faster than any neurodegenerative disorder placing greater demand on health care services [2]. Patients with PD require more care [3], that is progressively more complex than individuals without PD [4]. PD care can involve multiple specialties including neurologists, family physicians (FP), PD nurses, and diverse allied health professionals including physiotherapists and speech therapists. Accessing these services is critical for optimizing functioning in PwP given the wide range of symptoms that impact quality of life [5].

In Canada, the total cost for PD is estimated at nearly $1.2 billion as of 2020 due to health care expenditures for prevention, detection and treatment of PD, and indirect costs associated with reduced productivity due to disability and premature death [6]. Despite the universality, portability, and accessibility of the Canadian health care system through provincial health insurance programs as per the Canada Health Act’s principles [7], there is an uneven distribution of physician supply, which has led to reduced access and quality of care in some geographical areas compared to others [8–10]. Specialists tend locate in urban centres, and compared to FP in urban areas, those in rural areas may offer a broader range of services, including working in hospitals and emergency departments, and they may need to see patients from outside their communities [8]. As a result, people living in rural and remote areas face more difficulties accessing care due to the limited availability of specialists, longer time travel to access care, and physician turnover, which can lead to poorer health outcomes [9, 10]. In addition, access to neurologists is limited due to long wait times (two to three years), which can lead to delayed diagnoses [1]. Despite the burden of PD and observed disparities in health care access, there is limited evidence on the geographical accessibility of PD care in Canada and specifically in Ontario (for the purpose of this study). Better understanding how the patterns and determinants of PD-related health services use is essential for informing targeted programs and resource allocation for populations and areas in need of more support.

Operationally, access to health care can be defined as “the opportunity to reach and obtain appropriate health care services in situations of perceived need for care” [11]. This broad conceptualization is comprised of discrete aspects of the health care seeking process (potential access) or actual service use (realized access), whereby access is determined by characteristics of individuals, households, and neighbourhoods (demand side features), as well as health systems and providers (supply side features) [11]. Reported determinants of access to care include: system characteristics such as service costs, service capacity and distance to services; characteristics of individuals including age, income and ethnicity; and characteristics of neighbourhoods such as residential stability, levels of poverty and levels of education [12–17]. While the aforementioned determinants can be measured at the community or ecological level, this research does not explore other potentially important individual factors, such as personal health literacy, personal support networks, socialization, and participation in support groups, among others.

Our understanding of access to PD care has evolved in recent years, particularly within a U.S. context. A review of previous studies identified several barriers to care for PwP including personal-level barriers (skills required to seek healthcare services, ability to engage in healthcare, and cost for services) and system-level barriers (unavailability or inappropriate delivery of health care resources) [18]. One study identified important barriers to access to mental health care among PwP, including out-of-pocket costs, a lack of local services, transportation and trust in doctors, and physical impairments [19]. Several studies also found gender and racial disparities in access to PD care [20–23]. For example, one study found that the duration from symptom onset to a specialist visit was significantly longer for women than men [20]. Authors suggested that the findings may be explained by multiple factors beyond differences in disease progression and family history, including a reduced likelihood to emphasize symptoms during medical examinations, misdiagnoses due to the large prevalence of non-specific or non-motor PD symptoms, or physicians bias (i.e. physicians having predisposed perceptions that PD is more likely to occur in men). Reports on racial disparities in access to PD care suggested that compared to non-African Americans, African Americans were less likely to be treated, due to reduced access to medications or being seen with more advanced disease [21], had lower rates of medication use due to underdiagnosis, inadequate treatment, or biological factors [22], or had lower rates of surgery due to limited access to health insurance and specialist care, and disparities in cultural beliefs or socioeconomic status [23]. While these studies identified several factors influencing gender and racial disparities in access to PD care, they also suggested that these disparities are multifactorial, context-specific, and are not always fully explained by proposed factors.

To date, few studies have examined access to PD care in the Canadian context [4, 24]. A recent national survey of PwP, caregivers and health care providers revealed significant issues for PwP related to wait times and access to specialists and movement disorders clinics [24]. Reported barriers to care included long wait times, uninsured costs and lack of information. Hobson et al. (2012) conducted a retrospective case-control study using administrative health data, to examine rates of health care service use among PwP in the province of Manitoba [4]. Here, PwP who were older, lived in low-income areas, or lived in rural areas (where services are few) were less likely to access speciality services, but more likely to access family physician services. While they found that access and living in rural areas suggested that rurality/remoteness created geographic barriers to specialty care, a binary urban/rural classification did not account for the variability in spatial accessibility across Canada’s large rural and remote regions.

Spatial accessibility (i.e., geographic location of services relative to demand) is a critical component of access to care [11] that has received little attention in the context of PD. Commonly employed spatial accessibility measures include physician-to-population ratios using fixed-location boundaries [17] and measures of travel time or distance to services [25]. Problematically, physician-to-population ratios fail to incorporate movement across boundaries, or distance decay effects within boundaries; and measures of travel time or distance fail to capture actual supply and demand of services [26–28]. To address these limitations, various Floating Catchment Area (FCA) methods have been developed that incorporate the distribution of demand, supply, travel time, and increasingly, distance decay effects [12, 26, 27, 29]. To our knowledge, FCA approaches have not been applied in studies examining access to PD care.

Addressing the gaps in the existing PD access-to-care literature, and contributing to the larger international iCARE-PD project (https://icare-pd.ca/) goal of developing and implementing an improved PD care delivery model, this study has applied a geographic and socioeconomic lens within the context of Ontario, Canada to examine patterns and determinants of accessibility (realized access) to PD-related care. Specifically, the objectives of this study are to: (1) identify the spatial patterns of health services utilization; and (2) examine spatial and aspatial factors associated with individuals’ PD-related health services utilization. Understanding the accessibility to PD care is critical for the development of a more cost-effective and equitable system that can contribute to improving quality-of-life for PwP and their caregivers.

## Methods

### Design and setting

We conducted a retrospective, population-based study of individuals 60 year of age and over with physician-diagnosed PD or Parkinsonism (PKM) as of March 31, 2018, using administrative health data for the province of Ontario, Canada. PD and PKM patients are expected to have similar care needs [30]. Cases were identified by means of a previously validated case definition of 2 physician billing claims separated by at least 30 days over a 1-yr period, using physician diagnosis code 332 from the Ontario Health Insurance Plan (OHIP) database [30]. This case definition has a reported sensitivity of 70.6%-72.3% and a specificity of 99.9%-99.8% in adults and seniors, when compared with clinical evaluation by a physician. We used health services use information that is captured in administrative health databases securely held at ICES, formerly known as the Institute for Clinical Evaluative Sciences. ICES is a prescribed entity under section 45 of Ontario’s Personal Health Information Protection Act. Section 45 authorizes ICES to collect personal health information, without consent, for the purpose of analysis or compiling statistical information with respect to the management of, evaluation or monitoring of, the allocation of resources to or planning for all or part of the health system. Projects conducted under section 45, by definition, do not require review by a Research Ethics Board. This project was conducted under section 45, and approved by ICES’ Privacy and Compliance Office.

### Study area

**Fig 1:**
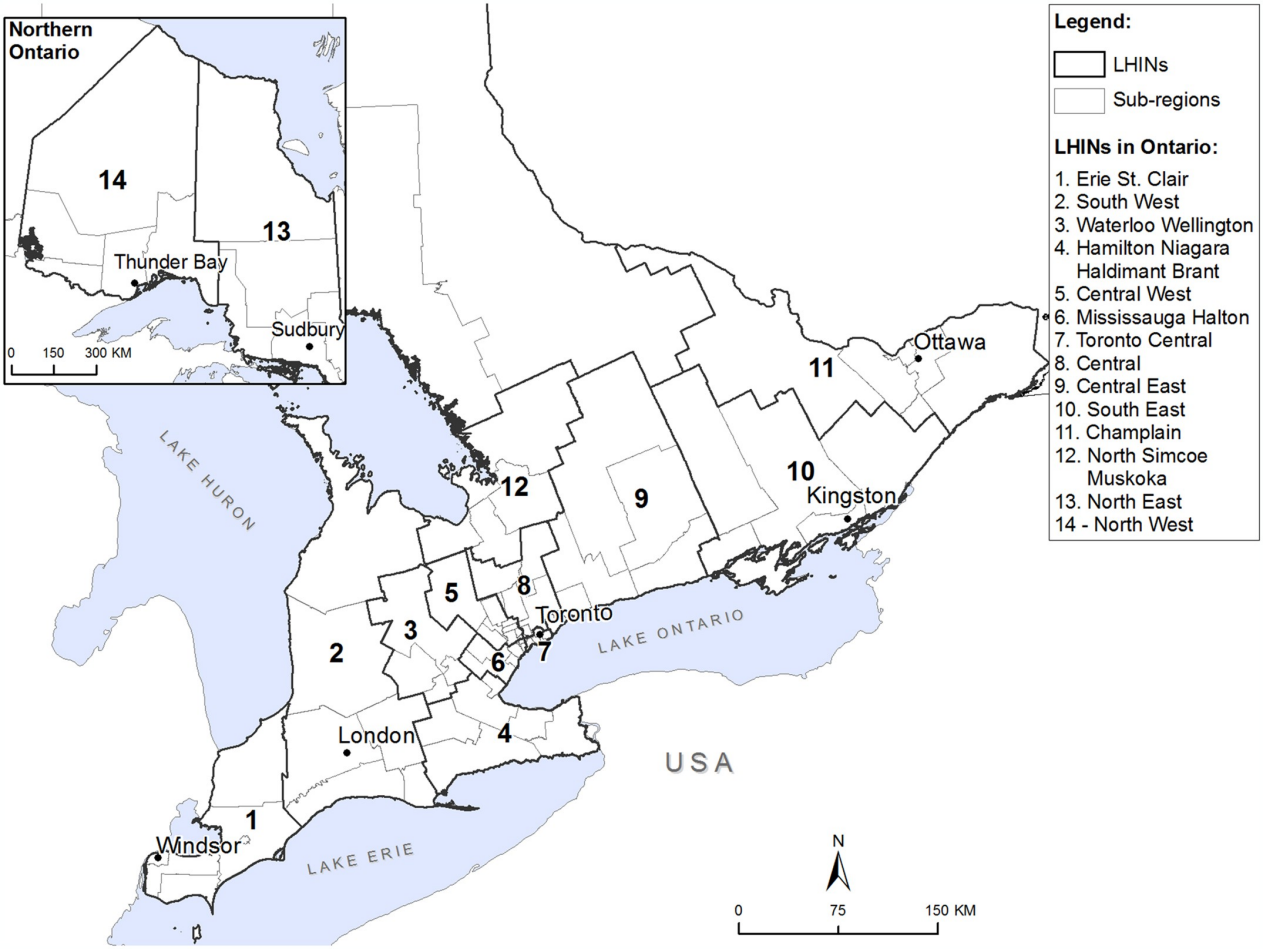
Study area, Local Health Integration Network (LHIN) sub-regions, Ontario, Canada.

The Province of Ontario has an area of over 1 million square kilometers and a population of approximately 14.3 million residents [31]. Northern Ontario is the most sparsely populated area in the province, while Southern and Eastern Ontario includes both sparsely populated rural agricultural areas and the province’s major urban centres (e.g., Toronto, Ottawa, Hamilton, Windsor). Ontario is divided into 14 administrative units called Local Health Integration Networks (LHINs) that are subdivided into 76 sub-regions (Fig 1). The median population size of LHIN sub-regions is approximately 140,000, with each sub-region having at least one acute care hospital, 150 primary care practices on average, and home and community care service providers [32]. LHIN sub-region populations range from approximately 7,000 in James and Hudson Bay Coasts (North East LHIN), in the North of the Province, to over 5 million in Western York Region (Central LHIN), in the South. For residents of Ontario, access to primary physician and hospital care services is universal through the Ontario Health Insurance Program (OHIP), although allied health services such as physiotherapy or speech therapy are not covered, and northern and rural residents must frequently travel significantly longer distances for in-person health care services as compared to their urban counterparts.

### Data sources

Data were obtained from de-identified administrative health databases held at ICES. De-identified datasets were accessed between January 15, 2020 and August 12, 2022 for research purposes, and linked using unique encoded identifiers and analyzed at ICES. In order to provide a complete health services profile for each individual, four main health services databases were linked at the individual level: (1) the Registered Persons Database (RPDB), which contains demographic information, location of residence and date of death; (2) the Canadian Institute of Health Information Discharge Abstract Database and Same Day Surgery (CIHI DAD-SDS), which contains information on all discharges from acute care hospitals and same day surgeries; (3) the OHIP Physician Claims database, which contains information on services provided by fee-for-service physicians and "shadow-billings" for physicians paid under alternate payment plans; and, (4) National Ambulatory Care Reporting System (NACRS) database, which contains information on patient visits to Emergency Departments. Other data sources used included the ICES Physician database (IPDB) to identify physician’s specialities and office locations (based on postal codes), ICES-derived Dementia database to identify individuals with physician-diagnosed dementia [30] and, geospatial files for mapping and spatial analysis including the Ontario LHIN sub-region boundaries from the Ministry of Health and Long Term Care (MOHLTC), dissemination areas from Statistics Canada, and Road Network files from DMTI Spatial Inc. [33].

### Dependent variables

We examined the number of PD-related physician office visits (using OHIP billing code 332) and hospitalizations (using ICD-10: G20, G21.0–0.4, G21.8–9, G22, F02.3) within 5 years prior to March 31, 2018 (i.e. between March 31, 2013 and March 31, 2018). Physician visits were broken down by physician specialty: neurologist, family physician (FP), and a combination of all selected providers of interest for PD care (i.e., emergency medicine, geriatric medicine, family physician, internal medicine, neurology, ophthalmology, physical medicine and rehabilitation, psychiatry, and urology).

### Independent variables

Area level and individual level independent variables were used in this analysis, as described in S1 Table. Area level variables were derived from RPDB and Ontario Marginalization Index (ON-MARG) 2016. RPDB was used to capture rurality based on Statistics Canada’s definition (i.e. living in communities with less than 10,000 population) [34]. ON-MARG was used to define area level socioeconomic and community characteristics associated with health care service utilization. ON-MARG is a geographically derived measure that incorporates socioeconomic variables drawn from the Canadian census [35]. This index was created using principal components analysis (PCA), and contains four dimensions of marginalization, namely material deprivation (inability to access and attain basic material needs), residential instability (family or housing instability), ethnic concentration (recent immigrant and/or visible minority), and dependency (no income from employment). For each geographic area, each dimension is expressed as standardized component scores, which are ranked and categorized into quintiles. Quintile 1 represents areas with the least marginalization and quintile 5 represents areas with the most marginalization. This index has been found to be stable, and associated with various health outcomes [36]. In this study, we excluded the dependency dimension from the analyses as it referred to population workforce eligibility, rather than a dependency on others due to needs for support/care or mobility factors. Area level independent variables were assigned to individuals based on their Dissemination Area (DA) of residence. In Ontario, for 2016, there are 20,160 DAs and each contains between 400 and 700 individuals (approximately 250 households). Individual level independent variables were derived from the health administrative databases described above, and include sex and age as of March 31, 2018, having a diagnosis for dementia prior to March 31, 2018, PD/PKM longevity (years living with PD/PKM) and comorbidities measured using the Charlson Index in the 5 years prior to March 31, 2018 [37]. These individual and area level variables were previously identified as being associated with health services utilization [11, 19, 38–42].

### Spatial accessibility

An enhanced 2-step floating catchment area (E2SFCA) method [12, 26, 27, 29] was employed to calculate physician-to-population ratios (PPR) within sequentially overlapping floating catchment areas. In the first step in traditional non-enhanced 2SFCA analysis, network distance/time buffer zones (also known as catchments), are placed around a point of health care supply, and provider-to-population ratios are calculated within the buffer(s). In the second step, buffers are placed around each point of population demand (i.e., each patient/potential patient), and the ratios from all provider points within that patient-based buffer are used to calculate access ratios values. The size of the catchment is determined by a choice of maximum travel time using road network distance where all services (or populations) within that catchment are considered accessible, and all locations outside are not. Problematically, this method assumes equal accessibility for each patient within the catchment area [43] and so excludes any providers and PD/PKM that lie just outside the buffer area. To address the hard-boundary of catchments using buffers, we adopted an enhanced 2SFCA method (or E2SFCA). This method involves applying a distance decay function to account for decreasing access as travel time increases within catchment areas [43]. The main advantage here is that no provider/patient points are excluded but those with travel times that are longer have a very small influence on provider-to-population ratios. Thus, in the E2SFCA method, the catchment boundaries are fuzzy rather than discrete.

**Fig 2:**
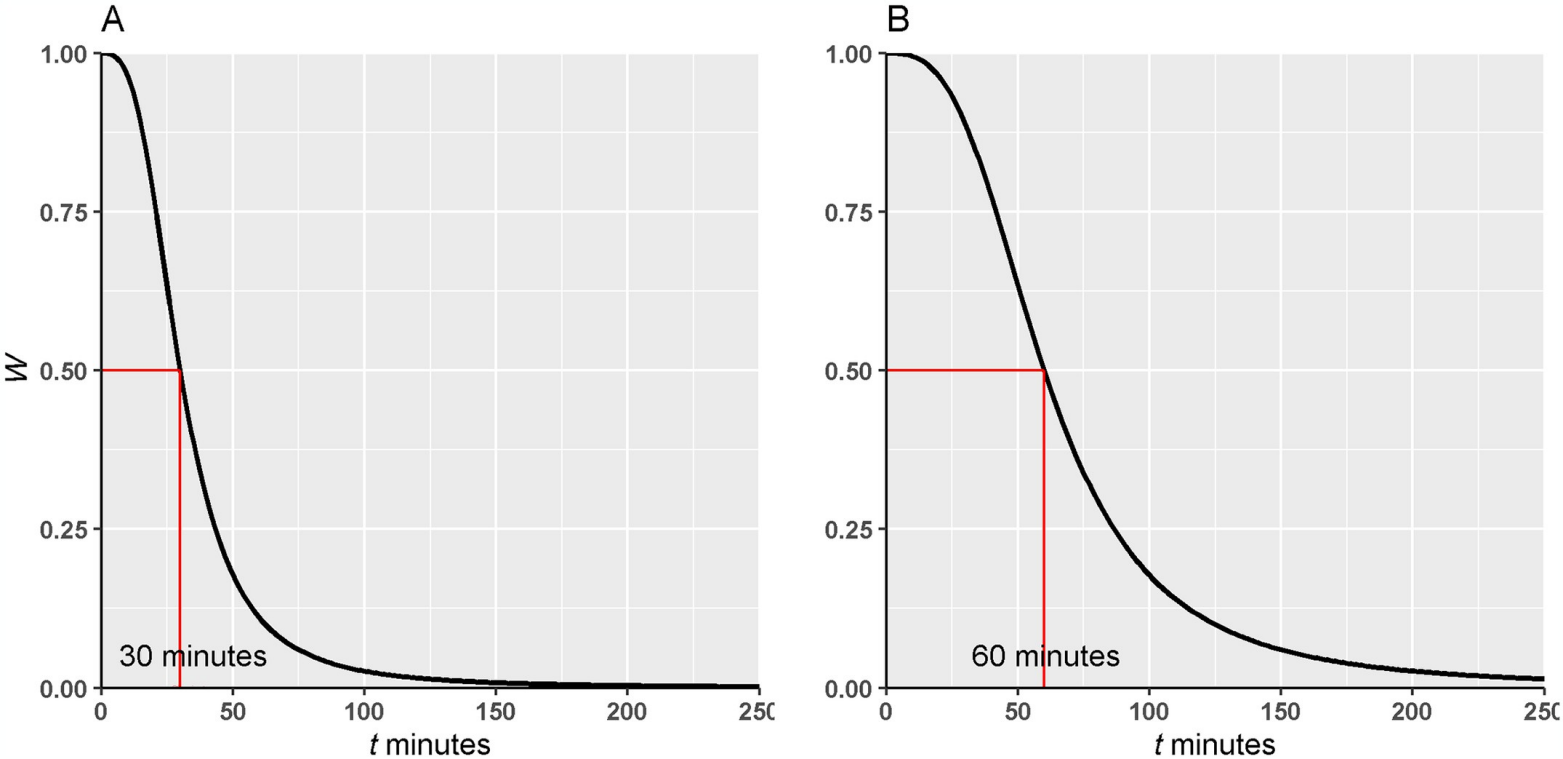
Changes in sigmoidal weighting as a function of the catchment travel minutes (*m*) based on Eq (1). Panel A) illustrates a catchment size defined by a midpoint, *m* = 30, yields a weight of *W* = 0.5, whereas in panel B), the larger midpoint of *m* = 60 assumes that a PwP would be willing to travel longer for care with their motivation to travel halved at 60 minutes.

The calculation of E2SFCA values involved capturing data for all PwP and visits to their care providers (i.e., FP, neurologists, and all selected providers) over the 5-year study period. A maximum travel time of 60 minutes was chosen to define the size of the fuzzy catchment and a sigmoid distance decay function was applied as the assumed model of inclusion for a PwP with respect to a provider or vice-versa. For example, for a single health provider’s catchment to compute the supply to demand ratio, *A for* a single health care provider:

$$\begin{array}{l} A=S/D\\ D=\sum_{i=1}^{n}W_{i}pwp_{i}\\ W={\left(1+{\left(\frac{t}{m}\right)}^{s}\right)}^{-1} \end{array}$$ (1)

where *S* is the supply or number of generalists/specialists at a given provider’s location and *D* is the demand or the number of PwP within the catchment travel-time of *S* weighted by *W*, which is the network-based minimum travel time-weighted value for a PwP. The term *pwp* represents a PwP within the catchment of the given provider and *pwp* = 1 ∀ PwP since each *pwp* counts as one occurrence of a PwP within the catchment. Thus based on *W*, the value of *W*_*i*_*pwp*_*i*_ will range effectively between 0 to 1 depending on the minimum time of travel, *t*, from the PwP’s location to the provider. The change in *D* as a function of *t* is dependent on the midpoint, *m*, and spread *s* which controls the steepness of the decay in *W* as a function of *t*. We consider this weighting function as a proxy for the motivation of a PwP to travel to the nearest health care provider (e.g. specialist, general practitioner) based on how long the travel takes when driving. For example, when *m* ≥ 30 minutes (in an urban region) then *W* would indicate a high motivation at shorter distances that declines at an increasing rate to 0.5 at thirty minutes and then at a decreasing rate slowly towards zero with little to no motivation beyond 60–120 minutes (Fig 2A). Likewise, whereas with *m* = 60, motivation is halved at 60 minutes travel time and closes toward zero after 120 minutes (Fig 2B).

The sigmoid model is a type of distance weighting model that assumes a person with Parkinson’s (PwP) is generally willing to travel for up to 60 minutes to reach health care services, a standard approximation used in Two-Step Floating Catchment Area (2SFCA) analyses. Unlike other models that might exclude anyone living more than 60 minutes away from care, the sigmoid model of distance decay shows a gradual decrease in motivation for PwP to travel longer distances than 60 minutes. So, for those living 70 minutes away, for example, there is still a willingness to travel to their closest health care service. However, as the travel distance increases significantly beyond this point, the likelihood of a PwP considering the health care service accessible drops drastically, with the distance weight nearing zero.

In rural areas a 60 min threshold, even with the sigmoidal accessibility model, would leave numerous patients with no motivation to access physicians. Rural patients have been shown to be willing to travel further to their nearest provider compared to urban patients [44]. As such, if no provider was found within the 60 minutes threshold in rural locations, we chose the travel time to nearest provider as the midpoint of our Sigmoidal function assuming that those in rural areas are used to travelling further than those in urban areas for access to the same amenities. Thus, our application of the E2SFCA method is regionally adaptive.

**Fig 3:**
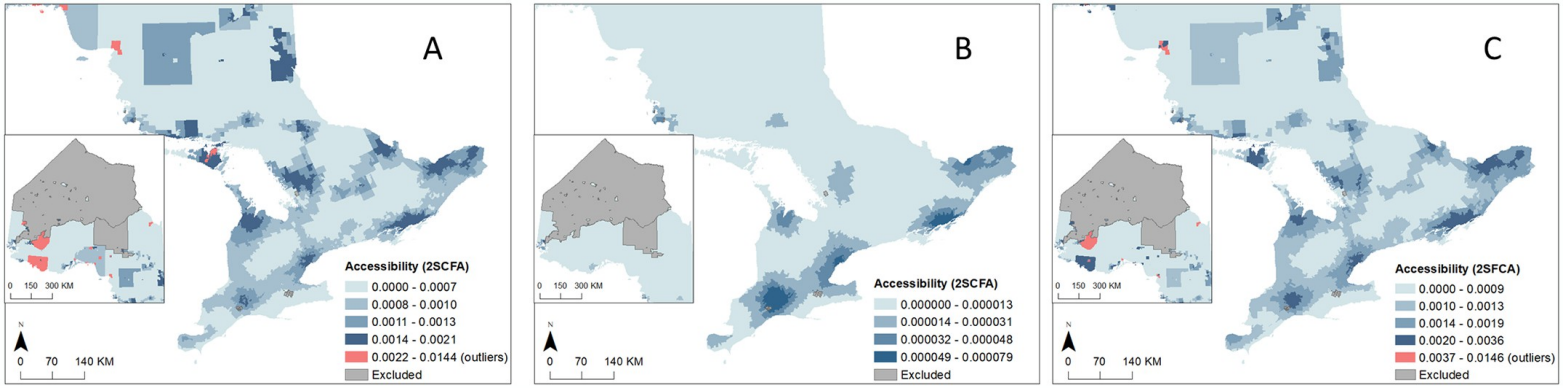
Variation of spatial accessibility values based on the enhanced 2-step floating catchment area (E2SFCA) method. Spatial accessibility values are presented for A) FP, B) neurologists, and C) all selected providers. All selected providers are comprised of the following: emergency medicine, geriatric medicine, family physician, internal medicine, neurology, ophthalmology, physical medicine and rehabilitation, psychiatry, urology.

Travel times were calculated using the DMTI 2018 Road Network [33]. We did not consider turns, one-way streets or elevation (under/overpasses) in our network which will have very minor effects on measures of spatial accessibility in urban areas. The accessibility values from this analysis by PD/PKM health care type are presented in Fig 3.

Spatial accessibility values were assigned to each person at the DA level. Continuous values were categorized using quintiles, with 1 indicating ‘lowest accessibility’ and 5 indicating ‘highest accessibility’. Sixty-one individuals (0.2%) were excluded from the study due to missing values associated with road network connectivity. Accessibility is greatest for all regions for FP, followed by all providers and then neurologists in general. Accessibility to neurologists in northwestern Ontario is centered around Thunder Bay and Kenora (Fig 3).

### Analyses

Analyses were conducted in 2 phases corresponding to the stated objectives. Phase 1 centered on the spatial analysis of disease prevalence and health service use by FP, neurologists, all selected providers combined and hospitalizations, at the LHIN Sub-Region level (Fig 1) to identify regional differences in age-standardized disease prevalence and service utilization rates by service type. Small numbers of PD/PKM cases in some areas limited our analysis to relatively large geographic units. Location of residence of PD/PKM patients were determined using patient postal codes. Comparative morbidity figures (CMFs) [45] were calculated for each outcome (i.e. physician visits to FP, neurologists, and all selected providers). CMFs are the ratio of the directly observed standardized rate in a given LHIN sub-region to the expected provincial rate. A CMF less than or greater than 1.0 indicates that the rate is below or above the provincial average, respectively. Confidence intervals (95%) for CMFs were calculated using the gamma method and mapped.

Phase 2 analysis involved individual-level, multivariate negative binomial regression modelling to assess the association between the dependent variables, PD-related health services utilization (i.e. number of encounters per day) by service type, and independent individual level and area level variables. We excluded PD-related hospitalizations from the dependent variables as there were too many patients (73.1%) with no hospital encounters. Statistical tests (Wald Statistic) were run for each possible interaction for all variables in the models. Terms that were significant (p<0.001) and theoretically justifiable, were included in the final models. The amount of collinearity in the models was assessed using spearman rank correlation test and Variance Inflation Factor (VIF) analysis, which found no strong collinearity. The use of negative binomial regression modelling is appropriate for count data that is over-dispersed, that is when the conditional variance exceeds the conditional mean [46]. It can be considered as a generalization of Poisson regression since it has the same mean structure as Poisson regression, and it has an extra parameter to model the over-dispersion. Here the distribution of count outcomes showed a presence of over-dispersion (i.e. variance > mean). All analyses were carried out in SAS version 9.4 (SAS Institute, North Carolina) and ArcGIS (v. 10.4).

## Results

A total of 35,482 individuals in Ontario were identified as prevalent PD/PKM cases as of March 31, 2018. The provincial prevalence rate of PD/PKM among those aged 60 or older is 10.7 per 1000 population. Within this cohort, over half were male with a small proportion living in rural areas (Table 1). The majority had no diagnosed comorbid conditions (Charlson index = 0) and almost a third were diagnosed with dementia.

**Table 1:** Characteristics of study population.

Individual characteristics	Total (N = 35,482)
Age (years), mean ± SD	77.2 ± 9.0
PD/PKM longevity (years), mean ± SD	7.5 ± 6.2
Sex (male), n (%)	15,763 (55.6%)
Residential instability^1^ quintiles, n (%)	
1 (lowest instability)	5,403 (15.2%)
2	5,926 (16.7%)
3	6,478 (18.3%)
4	7,059 (19.9%)
5	10,485 (29.6%)
Material Deprivation^1^ quintiles, n (%)	
1 (lowest deprivation)	8,057 (22.7%)
2	7,447 (21.0%)
3	6,809 (19.2%)
4	6,649 (18.7%)
5	6,389 (18.0%)
Ethnic Concentration^1^ quintiles, n (%)	
1 (lowest concentration)	6,930 (19.5%)
2	6,975 (19.7%)
3	6,506 (18.3%)
4	6,978 (19.7%)
5	7,962 (22.4%)
Rural residence, n (%)	3,745 (10.6%)
Charlson comorbidity index	
0 (no comorbidities)	23,428 (66.0%)
1 (low comorbidity score)	5,172 (14.6%)
2	3,413 (9.6%)
3+ (high comorbidity score)	3,469 (9.8%)

^1^ Subtotals vary from N = 35,482 owing to 131 (0.4%) missing cases.

Abbreviations: n, number; SD, standard deviation.

**Fig 4:**
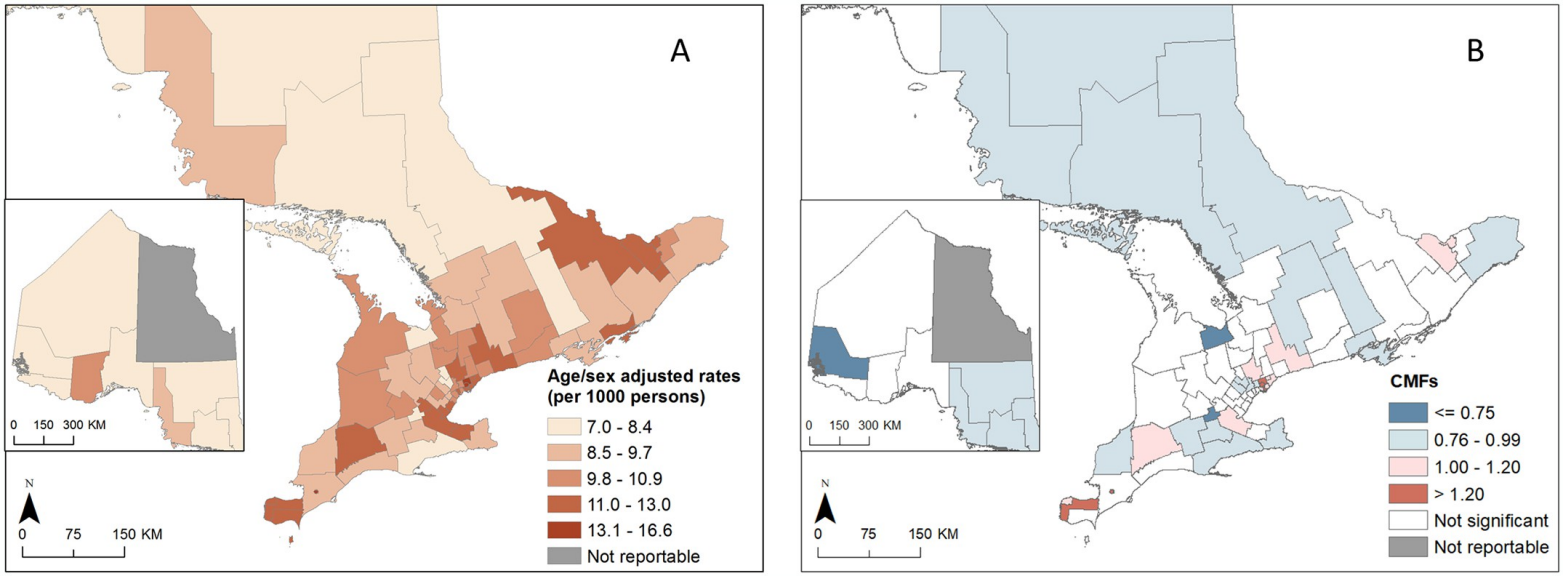
Age and sex adjusted PD/PKM prevalence rates and comparative morbidity figures (CMFs) in population aged 60 or older, Ontario, 2013–2018. Panel A) shows prevalence rates, and panel B) shows CMFs.

PD/PKM prevalence varied from 7.0 to 16.6 per 1000 between LHIN sub-region (Fig 4A). The highest prevalence was seen in urban areas including Windsor (Erie St. Clair LHIN), Ottawa (Champlain LHIN), and the LHINs around the city of Toronto. The CMF map (Fig 4B) showed these areas to be significantly high at 1.2 times the provincial average (CMF>1.2; p< 0.01). The lowest CMFs were found in rural parts of the province, most notably within the Northwest LHIN and North Simcoe Muskoka and Waterloo Wellington LHINs sub-regions where rates were 1.3 times below the provincial average (CMF <0.75; p<0.01). One northern LHIN sub-region was not reportable due to small counts.

**Fig 5:**
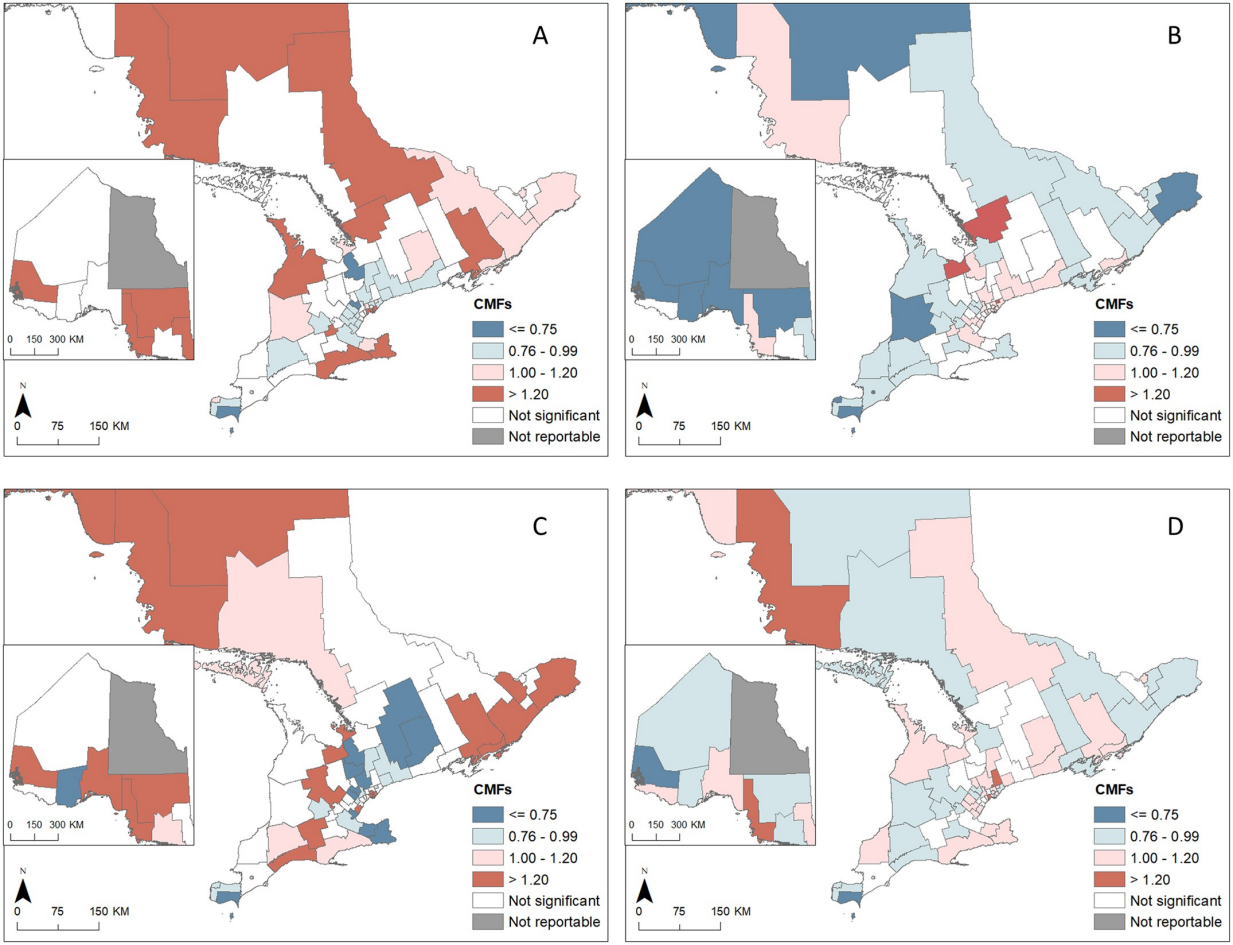
Comparative morbidity figures (CMFs) for PD/PKM related visits in Ontario, 2013–2018. Panels illustrate A) visits to FP, B) visits to neurologists, C) hospitalizations, and D) visits to selected providers.

Significantly high (p<0.05) CMFs for FP were seen across northern LHIN sub-regions and in more rural parts of southern Ontario including in parts of the South East and South West LHINs, where rates were more than 1.2 times the provincial average (Fig 5). CMFs were significantly low for more urban and southern LHINs, including, Central, Erie St. Clair and North Simcoe Muskoka where rates were approximately 1.3 times below the provincial average. CMF patterns for hospitalizations were similar to those for FP, although significantly low CMFs were seen in rural areas of the Central West LHIN and around the City of Thunder Bay in the North West LHIN.

Patterns of CMFs for Neurologists and All selected providers were similar. Significantly high CMFs were found mostly in urban and southern areas of the province that included the cities of Toronto, Hamilton and Niagara Falls (predominately urban region known as the Golden Horseshoe), as well as around the City of Thunder Bay in the North, where rates were often more than 1.2 times the provincial average. Significantly low CMFs were seen in more rural and remote LHINs in the North and East of the province as well as the southern sub-region near the city of Windsor.

Adjusted relative risk (aRR) and 95% confidence intervals for the multivariate negative binomial regression models are presented in Table 2. For the FP model, factors significantly associated with more frequent FP visits included living in the highest spatial accessibility areas (relative to lowest, aRR: 1.11; CI:1.04–1.18), and living in rural areas (relative to urban, aRR: 1.13; CI: 1.05–1:20). Factors significantly associated with less frequent FP visits included living in the second lowest spatial accessibility areas (relative to lowest, aRR: 0.90; CI: 0.85–0.96), living in high ethnic concentrations areas (e.g., highest ethnic concentration relative to lowest, aRR: 0.86; CI: 0.81–0.92), being older (for each additional year of age, aRR: 0.99; CI: 0.99–0.99), being female (aRR:0.87; CI = 0.84–0.90), and living in most deprived areas as years living with PD/PKM increased (e.g. in highest deprivation areas relative to lowest = aRR: 0.97; CI: 0.96–0.98).

**Table 2:** Factors associated with PD/PKM related physician visits by provider type.

	Family Physicians (FP) model		Neurologists model		All selected providers^†^ model	
Characteristics	aRR	95% CI	aRR	95% CI	aRR	95% CI
**Spatial accessibility**						
1 (lowest accessibility)	1.00	-	1.00	-	1.00	-
2	0.90****	0.85–0.96	1.04	0.99–1.08	0.99	0.95–1.03
3	0.98	0.92–1.04	1.19****	1.13–1.24	1.04*	1.00–1.09
4	0.99	0.93–1.05	1.14****	1.08–1.20	1.06**	1.01–1.10
5 (highest accessibility)	1.11**	1.04–1.18	1.10***	1.05–1.16	1.02	0.98–1.06
**Residential instability**						
1 (lowest instability)	1.00	-	1.00	-	1.00	-
2	0.95	0.89–1.01	1.03	0.99–1.08	1.00	0.95–1.04
3	1.01	0.95–1.08	1.06*	1.01–1.11	1.04	1.00–1.08
4	1.02	0.95–1.08	1.03	0.98–1.08	1.02	0.98–1.07
5 (highest instability)	1.03	0.97–1.10	1.01	0.97–1.06	1.02	0.98–1.06
**Ethnic concentration**						
1 (lowest concentration)	1.00	-	1.00	-	1.00	
2	0.91**	0.86–0.97	1.00	0.95–1.04	0.96	0.93–1.00
3	0.91**	0.85–0.96	1.03	0.99–1.08	0.98	0.94–1.03
4	0.91**	0.85–0.97	1.08**	1.03–1.13	1.02	0.97–1.06
5 (highest concentration)	0.86****	0.81–0.92	1.05*	1.00–1.11	0.98	0.93–1.02
**Rural vs. urban**	1.13****	1.05–1.20	0.89****	0.85–0.94	0.95*	0.91–1.00
**Age/years** (continuous)	0.99****	0.99–0.99	0.97****	0.97–0.97	0.98****	0.98–0.98
**Sex—female vs. male**	0.87****	0.84–0.90	0.89****	0.87–0.91	0.89****	0.86–0.91
**Interaction between PD/PKM longevity (years) and material deprivation**						
1 (lowest deprivation)	1.00	-	1.00	-	1.00	-
2	0.99*	0.98–1.00	1.00	0.99–1.01	1.00	0.99–1.00
3	0.98****	0.97–0.99	0.99**	0.98–0.99	0.98****	0.98–0.99
4	0.99**	0.98–1.00	0.98****	0.98–0.99	0.98****	0.98–0.99
5 (highest deprivation)	0.97****	0.96–0.98	0.97****	0.96–0.98	0.97****	0.96–0.97

aRR = adjusted relative risk; CI = confidence interval

* p<0.05;

** p<0.01;

*** p<0.001;

**** p<0.0001

^†^ All selected providers include emergency medicine, geriatric medicine, family physician, internal medicine, neurology, ophthalmology, physical medicine and rehabilitation, psychiatry, urology.

Note: Models controlled for comorbidities—Charlson Index [37], dementia; and variables in the interaction term–Parkinson disease/ Parkinsonism longevity (i.e. years living with Parkinson disease/ Parkinsonism) and material deprivation.

For the neurologists model, factors significantly associated with more frequent neurologist visits included: living in high spatial accessibility areas (e.g. highest spatial accessibility relative to lowest, aRR: 1.11; CI: 1.05–1.16), living in higher residential instability areas (relative to lowest, aRR: 1.06; CI = 1.01–1.11), living in higher ethnic concentration areas (relative to lowest, aRR: 1.08; CI: 1.03–1.13). Factors significantly associated with less frequent neurologist visits included: being older (for each additional year of age, RR: 0.97; CI: 0.97–0.97), being female (aRR:0.89; CI: 0.87–0.91), and living in most deprived areas as years living with PD/PKM increased (e.g. in highest deprivation areas relative to lowest, aRR: 0.97; CI: 0.96–0.98).

For the selected providers model, factors associated with more frequent visits to all providers included living in high spatial accessibility areas (e.g. medium-high spatial accessibility relative to lowest, aRR: 1.06; CI: 1.01–1.10). Factors significantly associated with less frequent visits to all providers included being older (for each additional year of age = aRR: 0.98; CI: 0.98–0.98), being female (aRR: 0.89; CI: 0.86–0.91) and living in most deprived areas as years living with PD/PKM increased (highest level of deprivation relative to lowest, aRR: 0.97; CI: 0.96–0.97).

## Discussion

There are several important results stemming from this analysis. We found significant spatial variation in PD/PKM prevalence and PD/PKM-related health services use across Ontario subregions. Higher rates for FP visits were found in rural southern areas and remote northern areas, and higher rates for prevalence, neurologist visits and all selected providers visits were found in urban areas. More frequent FP visits were associated with living in rural areas, whereas less frequent visits were associated with living in lower spatial accessibility areas, high ethnic concentration, being older, female, and living in more deprived areas as years living with PD/PKM increased. Neurologist visits were positively associated with living in high spatial accessibility areas and high ethnic concentration, and negatively associated with being older, female, and living in more deprived areas as years living with PD/PKM increased. Similar associations were found for all providers visits.

We found that PD/PKM prevalence rates were significantly below the provincial average in rural and northern areas, and significantly above the provincial average in urban areas. While this finding is consistent with a number of studies including geographical analysis of US Medicare data [47], others have reported higher rural rates, citing agricultural chemical exposures as an important risk factor [48, 49]. However, the Northern regions of the province are well outside the highest regions of agricultural intensity in southern and eastern Ontario. A more likely explanation for our findings relates to differential spatial access to specialist care and associated delays in diagnoses, rather than etiological factors [50, 51]. In Canada, a PD/PKM diagnosis must be confirmed by a neurologist, thus delayed diagnoses could be expected in rural areas where there is poor access to neurologists, leading to under-reporting of early-stages cases.

Our results also demonstrated significant spatial variability in services utilization, with higher rates of FP visits in rural and remote northern areas, where specialty services are unavailable, and higher rates of neurologist and all provider visits in well-serviced urban areas. The pattern is consistent with spatial accessibility patterns (Fig 3) and reinforces by our statistical models. In the neurologist model, spatial accessibility is positively associated with increased visits to neurologists, a relationship that is intuitive and well-established in the care access literature [4, 51, 52]. In the FP model, the relationship of spatial accessibility and service utilization is somewhat more nuanced. Here we found that those living in the second-lowest accessibility areas had less frequent FP visits compared to those in the lowest accessibility areas. This finding may be explained by an increased difficulty accessing physician’s offices in less accessible areas. It is also possible that patients in less accessible areas may rely on other ways of accessing FPs (which are not captured in this study) such as through telemedicine. The growth of telemedicine beyond remote areas during the COVID-19 pandemic may have broken down some of the spatial access barriers found herein [53]. While this has positive access implications for some, increased reliance on telemedicine creates new technological and communication barriers, particularly among the most elderly [54]. Future studies need to examine how COVID-19 has influenced the role of spatial accessibility on service use, as well as new technological and communication barriers that may be associated with telemedicine. Differences found between the effects of rurality and spatial accessibility on physician visits also suggest that rurality does not always reflect accessibility. This finding highlights the need to use more refined measure of geographical accessibility, perhaps one that takes into account the increased use of telemedicine together with sensitivity analyses using different functional forms (e.g. exponential, linear) that are used to represent motivation to travel and determination of such changes on analytical models to determine the robustness of conclusions around accessibility.

Demographic characteristics, namely gender and age, were both found to significantly influence service utilization. Women had lower number of physician visits than men in all three models. This has been reported previously [20, 55], explained by delays that women experience compared to men in obtaining accurate diagnoses and referrals to movement disorder and other specialists. Our findings may be explained by unmeasured confounding factors that may create gender differences in health services use, including the tendency to report health concerns or emphasize symptoms, the likelihood of being misdiagnosed, or the presence of physician bias [20, 21, 55, 56]. As for age, here we found that physician visits decreased with each additional year of age in all three models. Previous studies have suggested that as patients age, they may rely more on non-specialists care, home care or they may be admitted to long-term care homes [4, 57]. For example, Hobston et al. [4] found decreased visits to neurologists and other specialists, and suggested that as PwP age, medication regimes become simpler due to reduced tolerance, and care is transferred to non-specialists (i.e. FP) despite the increased complexity of PD/PKM care as the disease progresses. In our results, there was no corresponding increase in FP care, pointing to the possibility that PwP face additional barriers to all care as they age and the disease advances. In Zwicker et al.’s study [57], authors found that compared to those who died without PD/PKM, those who died with PD/PKM were more likely to be admitted to long-term care or receive more home care.

Community marginalization is significantly associated with services used, but the direction of the associations varied by the type of physician visit examined. PwP living in areas of high ethnic concentration had less frequent FP visits, but more frequent neurologist visits. This pattern is consistent with higher ethnic concentrations seen in Ontario’s urban centres where neurologist accessibility is high. This finding is inconsistent with PD studies in the U.S., that commonly show lower rates of neurologist visits among non-whites [58], a relationship that is attributed to delayed diagnosis and financial barriers to care. It is important to note that it is the influence of small-area ethnic concentration on service utilization that is examined here, not individual ethnic characteristics. Future research should examine the role of ethnicity at the individual level in determining access to PD/PKM care. Secondly, PwP residing in deprived areas had less frequent visits to FP, neurologists and all providers combined, as years living with PD/PKM increased. This finding is comparable to Hobson et al.’s (2012) study in Manitoba, Canada, although there, deprivation was measured with income alone, and no interaction was considered [4]. Our findings suggest that system level barriers (e.g., disparity in access to health care services across population groups, poor communication between patients and providers due to cultural of linguistic barriers) and community level barriers (e.g. transportation, community supports) may be more common in deprived areas, and increasingly difficult to overcome as autonomy, health status and self-efficacy decline as PD/PKM progresses [18]. This points to the importance of policies being responsive to the needs and evolving challenges faced by PwP as they navigate the health care system.

Our study had several strengths. We used large, population-based, administrative health data on health care encounters for Parkinson disease in Ontario. This allowed us to identify the PD/PKM population, which was sufficiently large to ascertain cases of this relatively rare condition. Further, we employed a sophisticated geographic accessibility measure using a distance decay function, which allowed us to account for the variation in accessibility with increasing travel time within each catchment. The use of a sigmoid model represents a better reflection of reality compared with non-enhanced 2SFCA methods, because the likelihood of a patient refusing to travel an extra 5, 10, 15 minutes to medical care is probably small on a ‘good’ day. This model did not factor in the many other issues that could affect willingness to travel a specified distance, such as seasonal variations in weather conditions, time of day, traffic conditions etc. In general, an individual’s destination choice involves several factors beyond travel time and thus in the non-enhanced 2SFCA, the hard *n*-minute boundary for travel is unlikely to represent true accessibility, demand or motivation to travel. The E2SFCA measure using a sigmoidal function addressed that problem by presenting a decreasing likelihood of travel with increasing travel time to the provider’s location. A person’s travel decision to a point of care, or otherwise, are complex and thus better modelled as a fuzzy process [59–61]. This sigmoidal function is however only a rudimentary decision function and removes only the binary boundary of 60 minutes while maintaining the idea that at some point a health care service becomes too far to be considered practical or accessible.

There were also a few limitations to this study. First, while the use of population-based administrative databases and a validated algorithm allowed us to identify the PD/PKM population in Ontario, it did not capture cases where no physician diagnosis occurred, uninsured services were accessed, or people did not seek care, which may represent a potential selection bias. While we were unable to capture those cases from the administrative databases used in this study, future studies may be needed to assess the possible impact of missing data on this study population. It is also possible that the validity of the PD/PKM prevalence and health services use measures may have been impacted by changes in legislations that have occurred during the study timeframe, such as the Ontario Patients Act in 2017. Second, the use of spatially aggregated data generally introduces a modifiable areal unit problem (MAUP) [62], and our analytical outcomes would be different if different areal units were used. Census data as well as LHIN and subgroups are pre-defined polygon geometries imposed on this research by the data availability. Thus, there is little that can be done to analyze these results at other geographic levels. PwP census information and the Ontario marginalization index values could only come from dissemination areas because these are continuous across Canada whereas Census Tracts, a higher level of spatial aggregation are only available in population centers and exclude rural and remote regions. The MAUP is an inherent limitation of all research that uses ecological level data. Second, other PD/PKM-related health services use, such as long-term care and home visits, were not included in this study due to the low number of visits found. For physician visits in long-term care, given that geographical access is not typically a consideration, we concluded that additional explorations were not necessary. Future studies may be needed however, to explore the impact of spatial accessibility on home care visits. In addition, we included FP and neurologist in “all selected providers” in this study to explore the geographical patterns and determinants of all health services use combined, including any specialty relevant to Parkinson disease; this method however, did not allow us to fully capture the differences across specialties. Fifth, the area-level socioeconomic status measures used in this study can only represent contextual level factors, not individual-level factors. For example, while results may be used to understand how low income communities might influence access, we cannot judge how individual income influences access as this would entail committing an ecological fallacy. There may also be other unmeasured factors, such as the differences in demand for services or costs of uninsured services, which may have influenced health services use for PwP. Finally, the influence of telemedicine on access was not considered here and thus, the relative importance of spatial accessibility may have been reduced in some contexts. However, telemedicine has introduced new barriers associated with the availability of communication technologies and related infrastructure (e.g. broadband), and has underlined the limited understanding of how to use these technologies, particularly among the elderly [54].

This research aimed to develop a better understanding of the spatial characteristics and determinants of PD/PKM health care use in Ontario. We highlighted the presence of spatial patterns of PD/PKM prevalence and health service use, and identified factors that influence accessibility to health services beyond disease characteristics. Results from this study may inform planning strategies by the iCARE-PD project for the development and delivery of self-management support, integrated and technology-enabled care to overcome poor access to care in rural Canada. In addition, our findings identified areas with better and poorer accessibility to health care for PwP which may inform health policies and resource allocation aimed at reducing social and spatial inequalities. With the growth of telemedicine during the COVID-19 pandemic and the increased use of other innovative virtual care networks, it is possible that these advancements have facilitated access to virtual specialist care for PwP and provided opportunities to connect FP and specialists to improve the support and scope of care services for FP in rural and remote areas. More research is needed to better understand how various health care delivery models may impact the care and health outcomes of PwP.

## Supporting information

S1 Table List of study variables. (DOCX)

S1 Checklist Human participants research checklist. (DOCX)
