# Supplementary material for: Patterns and determinants of health care utilization among people with Parkinson’s disease: A population-based analysis in Ontario, Canada
Source: PLoS One. 2024 Jun 21;19(6):e0305062. doi: 10.1371/journal.pone.0305062 (PMC11192415; doi:10.1371/journal.pone.0305062)
Supplement: S1 Table — (DOCX) [file pone.0305062.s001.docx]

**S1 Table. List of study variables**

| Variable | Data source | Codes/ Definition | Level of analysis | Model variable |
| --- | --- | --- | --- | --- |
| PD-related visits to family physicians | OHIP  IPDB | OHIP - Dx code: 332  IPDB - specialty: family physician | Individual | Dependent |
| PD-related visits to neurologists | OHIP  IPDB | OHIP - Dx code: 332  IPDB - specialty: neurologist | Individual | Dependent |
| PD-related visits to all selected providers visits | OHIP  IPDB | OHIP - Dx code: 332  IPDB - specialty: emergency medicine, geriatric medicine, family physician, internal medicine, neurology, ophthalmology, physical medicine and rehabilitation, psychiatry, urology | Individual | Dependent |
| PD-related hospitalizations | CIHI-DAD | ICD-10: G20, G21.0-0.4, G21.8-9, G22, F02.3 | Individual | Not applicable |
| Spatial accessibility | DMTI 2018 Road Network  RPDB  IPDB | Enhanced 2-step floating catchment area method [1] | Area | Independent |
| Marginalization indices:   - Material deprivation - Residential instability - Ethnic concentration | ONMARG | As defined in Matheson and Van Ingen (2016) [2] | Area | Independent |
| Rurality | RPDB | Communities with < 10,000 population [3] | Area | Independent |
| Age | RPDB |  | Individual | Independent |
| Sex | RPDB |  | Individual | Independent |
| Having a diagnosis for dementia | Dementia (ICES-derived dataset) | As defined in Jaakkimainen et al. (2016) [4] | Individual | Independent |
| PD/PKM longevity (i.e. years living with PD/PKM) | OHIP | OHIP Dx code: 332 | Individual | Independent |
| Charlson comorbidity index | CIHI-DAD | As defined in Charlson et al. (1987) [5] | Individual | Independent |

**Abbreviations:** CIHI-DAD, Canadian Institute of Health Information Discharge Abstract Database; Dx, Diagnosis; IPDB, ICES Physician database; ONMARG, Ontario Marginalization index; OHIP, Ontario Health Insurance Plan; PD, Parkinson’s disease; PD/PKM, Parkinson’s disease/Parkinsonism; RPDB, Registered Persons Database.

References**:**

1. Bauer J, Groneberg DA. Measuring Spatial Accessibility of Health Care Providers – Introduction of a Variable Distance Decay Function within the Floating Catchment Area (FCA) Method. PLoS One. 2016;11: e0159148. doi:10.1371/journal.pone.0159148

2. Matheson FI, Van Ingen. T. 2016 Ontario marginalization index: user guide. Toronto, ON: St. Michael’s Hospital. Public Health Ontario. 2018. Available: https://ontariohealthprofiles.ca/onmargON.php

3. du Plessis V, Beshiri R, Bollman RD, Clemenson H. Definitions of rural. Statistics Canada Rural and Small Town Canada Analysis Bulletin. 2001;3. Available: https://www150.statcan.gc.ca/n1/pub/21-006-x/21-006-x2001003-eng.pdf

4. Jaakkimainen RL, Bronskill SE, Tierney MC, Herrmann N, Green D, Young J, et al. Identification of Physician-Diagnosed Alzheimer’s Disease and Related Dementias in Population-Based Administrative Data: A Validation Study Using Family Physicians’ Electronic Medical Records. Journal of Alzheimer’s Disease. 2016;54: 337–349. doi:10.3233/JAD-160105

5. Charlson ME, Pompei P, Ales KL, MacKenzie CR. A new method of classifying prognostic comorbidity in longitudinal studies: Development and validation. J Chronic Dis. 1987;40: 373–383. doi:10.1016/0021-9681(87)90171-8
